# Prognostic significance of the inhibitory-to-stimulatory immune checkpoint ratio in patients with breast cancer

**DOI:** 10.3389/fonc.2025.1524861

**Published:** 2025-02-21

**Authors:** Chuangang Tang, Xiang Hu, Dawei Hao, Tao Chen, Pei Wang, Changwen Li, Chengling Chen, Yongcheng Li, Xiaowen Hao, Zeng Yuan

**Affiliations:** ^1^ Department of Breast Surgery, Xuzhou Central Hospital, The Affiliated Xuzhou Hospital of Medical College of Southeast University, Xuzhou, Jiangsu, China; ^2^ Department of Radiotherapy, Xuzhou Central Hospital, The Affiliated Xuzhou Hospital of Medical College of Southeast University, Xuzhou, Jiangsu, China; ^3^ The Xuzhou Clinical College of Xuzhou Medical University, Xuzhou, Jiangsu, China; ^4^ Department of Medical Oncology, Xuzhou Central Hospital, The Affiliated Xuzhou Hospital of Medical College of Southeast University, Xuzhou, Jiangsu, China; ^5^ Department of Obstetrics and Gynecology, Qilu Hospital of Shandong University, Shandong, China

**Keywords:** breast cancer, TCGA, prognosis, IDO1, TMIGD2

## Abstract

**Background:**

Accumulating evidences suggested that immune checkpoints (ICPs) played an important role in malignancies including breast cancer (BRCA). We aimed to investigate whether inhibitory-to-stimulatory immune checkpoint ratio (ISICPR) could be used as a prognostic marker for BRCA.

**Methods:**

BRCA patients were enrolled from The Cancer Genome Atlas (TCGA). Survival analysis was performed with Kaplan-Meier (KM) methods. X-tile was used to calculate the optimal cut-off values of ISICPRs. Univariate and multivariate Cox regression analysis were carried out to identify prognostic factors for BRCA patients. Tissue microarray was used to validate our findings.

**Results:**

In total, 586 BRCA patients were collected, including 104 cases of stage I, 330 of stage II, 139 of stage III, and 13 of stage IV. Univariate analysis showed that four ISICPRs (PDCD1/CD27 ratio, PDCD1/TNFSF4 ratio, IDO1/TMIGD2 ratio, and IDO1/TNFSF4 ratio) were significantly associated with the survival of BRCA patients. After adjusting for confounders, multivariate analysis indicated that only the IDO1/TMIGD2 ratio was an independent prognostic factor. The optimal cut-off values for the IDO1/TMIGD2 ratio were set at 4.4 and 6.3. Survival analysis indicated that the high-ratio group (ratio > 6.3) had a worse prognosis than both the low-ratio (ratio < 4.4) and medium-ratio group (4.4 < ratio < 6.3) (P < 0.001), which was further validated by BRCA tissue microarray.

**Conclusions:**

We found that IDO1/TMIGD2 ratio was an independent prognostic factor for BRCA. On one hand, dual targeting of IDO1 and TMIGD2 may be a more effective therapeutic strategy for patients with a high IDO1/TMIGD2 ratio. On the other hand, ISICPR was a promising indicator with high clinical values and worthy of further promotion in other cancers.

## Introduction

Breast cancer (BRCA) is the most common malignancy in women but is rare in men (1, 2). According to global cancer statistics, BRCA had the second-highest incidence worldwide (after lung cancer), with an estimated 2,088,849 new cases in 2018 (3). BRCA is characterized by remarkable heterogeneity and is broadly divided into four subtypes based on estrogen receptor (ER), progesterone receptor (PR), and human epidermal growth factor receptor-2 (HER2) status: luminal A (ER+/PR+/HER2–), luminal B (ER+/PR+/HER2+), HER2-enriched (ER–/PR–/HER2+) and triple-negative BRCA (ER−/PR−/HER2−) (4). Among the four subtypes, triple-negative BRCA generally carries the worst prognosis (5). Although luminal A BRCA usually has a favorable outcome, the occurrence of metastatic disease also leads to a poor prognosis. Therefore, there is an urgent need to further explore the molecular mechanisms underlying BRCA initiation and development and to search for new biological markers.

Accumulating evidence suggests that immunotherapies targeting immune checkpoints (ICPs) have exhibited promising therapeutic effects in malignancies including BRCA (6–8). ICPs can be divided into two major categories: stimulatory and inhibitory ICPs (9). Most research has focused on inhibitory ICPs including PDCD1. In the large clinical trial IMpassion130, Atezolizumab (a PD-1 inhibitor) plus nab-paclitaxel significantly improved the prognosis of PD-L1-positive metastatic triple-negative BRCA patients (10). The TONIC trial demonstrated that doxorubicin and cisplatin induction enhanced the antitumor immune response to PDCD1 blockade in triple-negative BRCA (11). In addition to the PD-1/PD-L1 pathway, other immune checkpoints such as CTLA-4 and LAG-3 have also garnered attention in BRCA (12–14). In contrast, fewer studies investigated the molecular mechanisms and clinical applications of stimulatory ICPs. A CD40L bystander vaccine successfully controlled BRCA cell growth in an *in vivo* animal study (15). These findings indicate that both stimulatory and inhibitory ICPs play important roles in maintaining immune balance. In line with this, we speculate that an imbalance in the inhibitory-to-stimulatory immune checkpoint ratio (ISICPR) may be a key indicator of tumor initiation and development.

In this study, we investigated whether ISICPRs could be used as prognostic markers for BRCA.

## Methods

### Prognostic evaluation of ISICPRs

BRCA patients were enrolled from The Cancer Genome Atlas (TCGA) (https://tcga-data.nci.nih.gov/tcga/), a large database that includes both clinical and gene expression data. The inclusion criteria were as follows: (1) Patients aged 18 years or older; (2) Patients with confirmed ER status, PR status, and HER2 status; (3) Patients with definite American Joint Committee on Cancer (AJCC) tumor-node-metastasis (TNM) staging information; (4) Patients with available gene expression data for candidate genes; and (5) Patients who were available follow-up information. Baseline clinical characteristics included age, gender, ER status, PR status, HER2 status, TNM stage, mRNA expression levels, survival time, and status. The study was conducted in compliance with local and federal regulations and was approved by the ethics committee of Xuzhou Central Hospital. Informed consent from patients was obtained by the TCGA consortium.

Common ICPs include CD274, CD276, CTLA4, HHLA2, ICOS, ICOSLG, PDCD1, PDCD1LG2, TMIGD2, VTCN1, BTLA, CD27, CD40L, CD40, CD70, TNFRSF18, TNFRSF4, TNFRSF9, TNFSF14, TNFSF4, TNFSF9, HAVCR2, IDO1, LAG3, FGL1, ENTPD1, NT5E, SIGLEC15, VSIR, NCR3 (16).

### Characteristics of key ISICPRs

Clinical characteristics between the low/medium IDO1/TMIGD2 ratio and high IDO1/TMIGD2 ratio groups were compared using the χ^2^ test and Fisher’s Exact test. Survival analysis was performed using Kaplan-Meier (KM) methods. The primary study endpoint was overall survival (OS), defined as the time from initial diagnosis of BRCA to all-cause death or the last follow-up. Univariate and multivariate Cox regression analyses were conducted to identify prognostic factors for BRCA patients. Age was treated as a categorical variable and divided into two groups based on the median age. Correlation analysis between IDO1 and TMIGD2 was performed using the GEPIA database (gepia.cancer-pku.cn/). Immune correlation analysis was conducted using the online TIMER database (https://cistrome.shinyapps.io/timer/).

### Validation of tissue microarray

The tissue microarray for BRCA was purchased from Youluze BioTech (China). The follow-up period ranged from 4 to 101 months, with 39 deaths occurring during the follow-up. It should be noted that 17 patients were lost to follow-up and were therefore excluded from the analysis. There was a total of 118 BRCA patients included. IDO1 (Cat No.: ab211017) and TMIGD2 (Cat No.: ab121333) antibodies were purchased from Abcam (Abcam, UK). The staining was scored by two independent pathologists, and discrepancies were further assessed by a third pathologist. The staining intensity was graded as follows: negative (−); low (+) and medium (++). No high staining (+++) was observed in the assessment. An IDO1^medium^/TMIGD2 ^low/negative^ was defined as a high ratio, and the other categories were defined as low/medium ratio, including IDO1^medium^/TMIGD2^medium^, IDO1^low/negative^/TMIGD2^low/negative^, and IDO1^low/negative^/TMIGD2^medium^.

### Statistical analysis

The statistical analysis was performed using SPSS (version 19.0). X-tile (Yale University, New Haven, CT, USA, version 3.6.1) was used to determine the cut-off values for ISICPRs. X-tile employs a recursive partitioning algorithm to identify the optimal cut-off points for continuous variables by maximizing the statistical significance of survival differences. In this study, all possible cut-off points within the range of ISICPR values were evaluated. *P < 0.05: indicates statistical significance; **P < 0.01: indicates high significance; ***P < 0.001: indicates extreme significance.

## Results

### Identification of key ISICPRs

**Figure 1:**
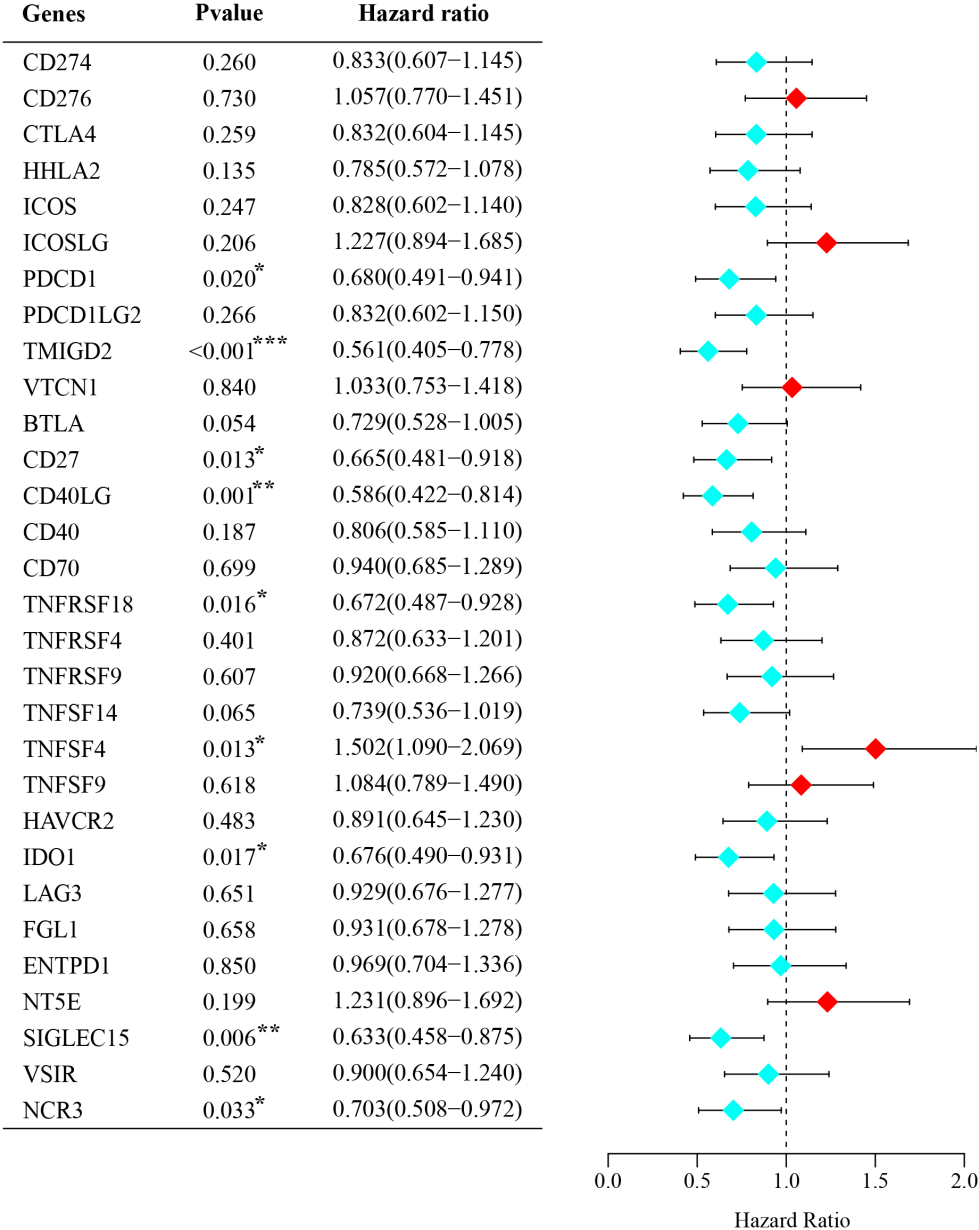
Forest plot of 30 common ICPs in BRCA. *P < 0.05; **P < 0.01; ***P < 0.001.

Using the TCGA database, we conducted a batch survival analysis of 30 common genes and identified 9 genes associated with the OS of BRCA patients (**Figure 1**), including TMIGD2, CD27, CD40L, TNFRSF18, TNFSF4, NCR3, PDCD1, IDO1, and SIGLEC15. Of these, TMIGD2, CD27, CD40L, TNFRSF18, TNFSF4, and NCR3 were classified as stimulatory ICP molecules, while PDCD1, IDO1, and SIGLEC15 were inhibitory ICP molecules. These 9 genes were then used to generate 18 potential ISICPRs, including PDCD1/TMIGD2, PDCD1/CD27, PDCD1/CD40L, PDCD1/TNFRSF18, PDCD1/TNFSF4, PDCD1/NCR3, IDO1/TMIGD2, IDO1/CD27, IDO1/CD40L, IDO1/TNFRSF18, IDO1/TNFSF4, IDO1/NCR3, SIGLEC15/TMIGD2, SIGLEC15/CD27, SIGLEC15/CD40L, SIGLEC15/TNFRSF18, SIGLEC15/TNFSF4, and SIGLEC15/NCR3.

**Figure 2:**
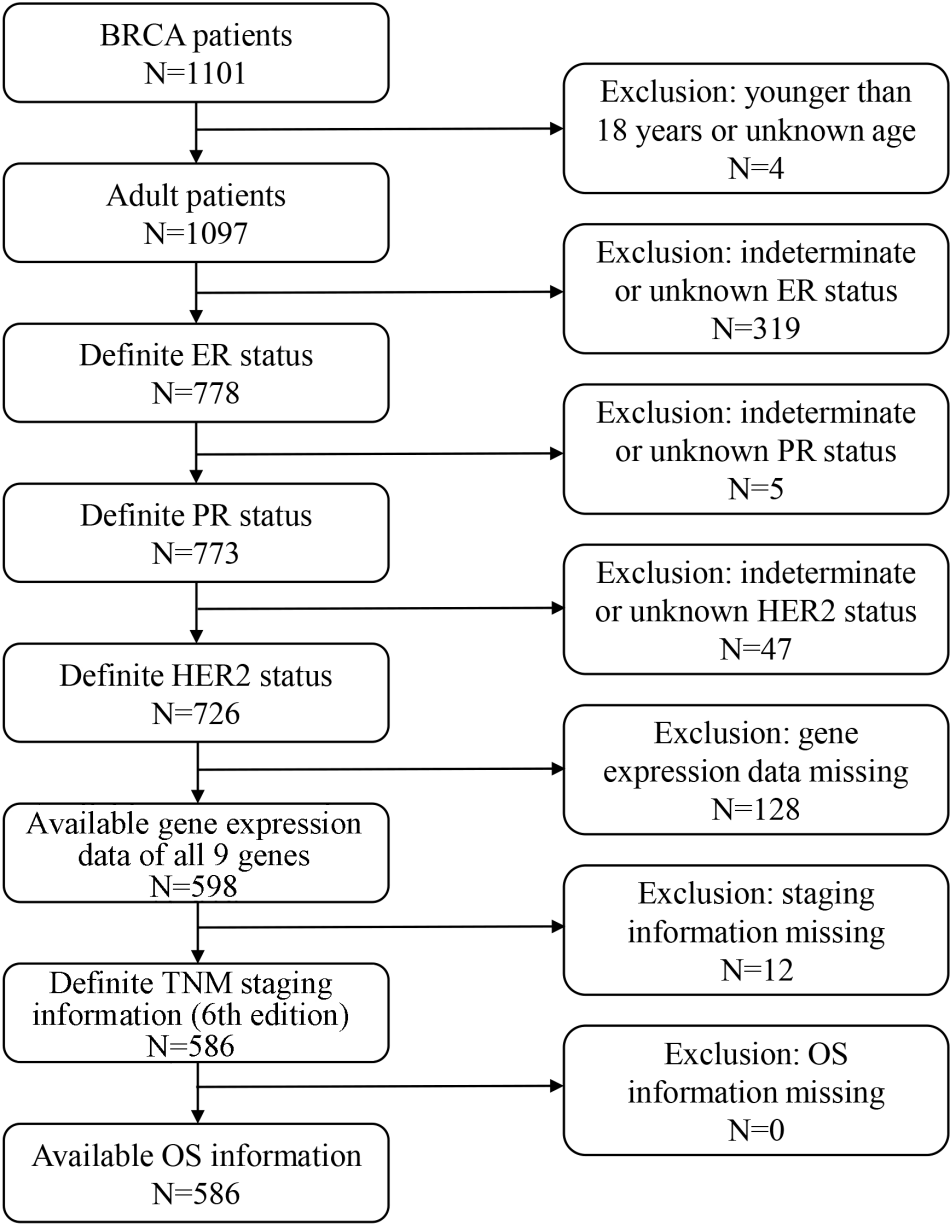
A flow-diagram of the screening procedure.

To assess the prognostic value of ISICPRs, a rigorous participant screening process was implemented (**Figure 2**). A total of 586 BRCA patients were included, with baseline clinical characteristics summarized in **Table 1**. The study cohort included participants aged between 26 and 90 years, with a median age of 57 years. The vast majority of patients were female (580, 99.0%) and had not undergone neoadjuvant treatment (577, 98.6%). Patients were distributed across clinical stages as follows: stage I (n=104), stage II (n=330), stage III (n=139), and stage IV (n=13). The initial weight of tumor ranged from 20 to 1,740g, with a median weight of 220g. In terms of biomarkers, 455 patients had ER-positive BRCA, 399 had PR-positive BRCA, and 89 had HER2-postive BRCA. During the follow-up period, 71 all-cause deaths were recorded.

**Table 1:** Baseline characteristics of entire cohort.

Variables	N
Total	586
Gender	
Male	6
Female	580
Age, yrs	
< 60	331
≥ 60	255
Median (range)	57 (26-90)
ER status	
Positive	455
Negative	131
PR status	
Positive	399
Negative	187
HER2 status	
Positive	89
Negative	497
TNM stage	
I	104
II	330
III	139
IV	13
Vital status	
Dead	71
Live	515
History of neoadjuvant treatment^a^	
No	577
Yes	8
Initial weight of tumor^b^, g	
Median (range)	220 (20-1740)

ER, estrogen receptor; PR, progesterone receptor; HER2, human epidermal growth factor receptor-2; TNM, tumor-node-metastasis. yrs, years; g, gram. ^a^one case missing. ^b^84 cases missing.

**Figure 3:**
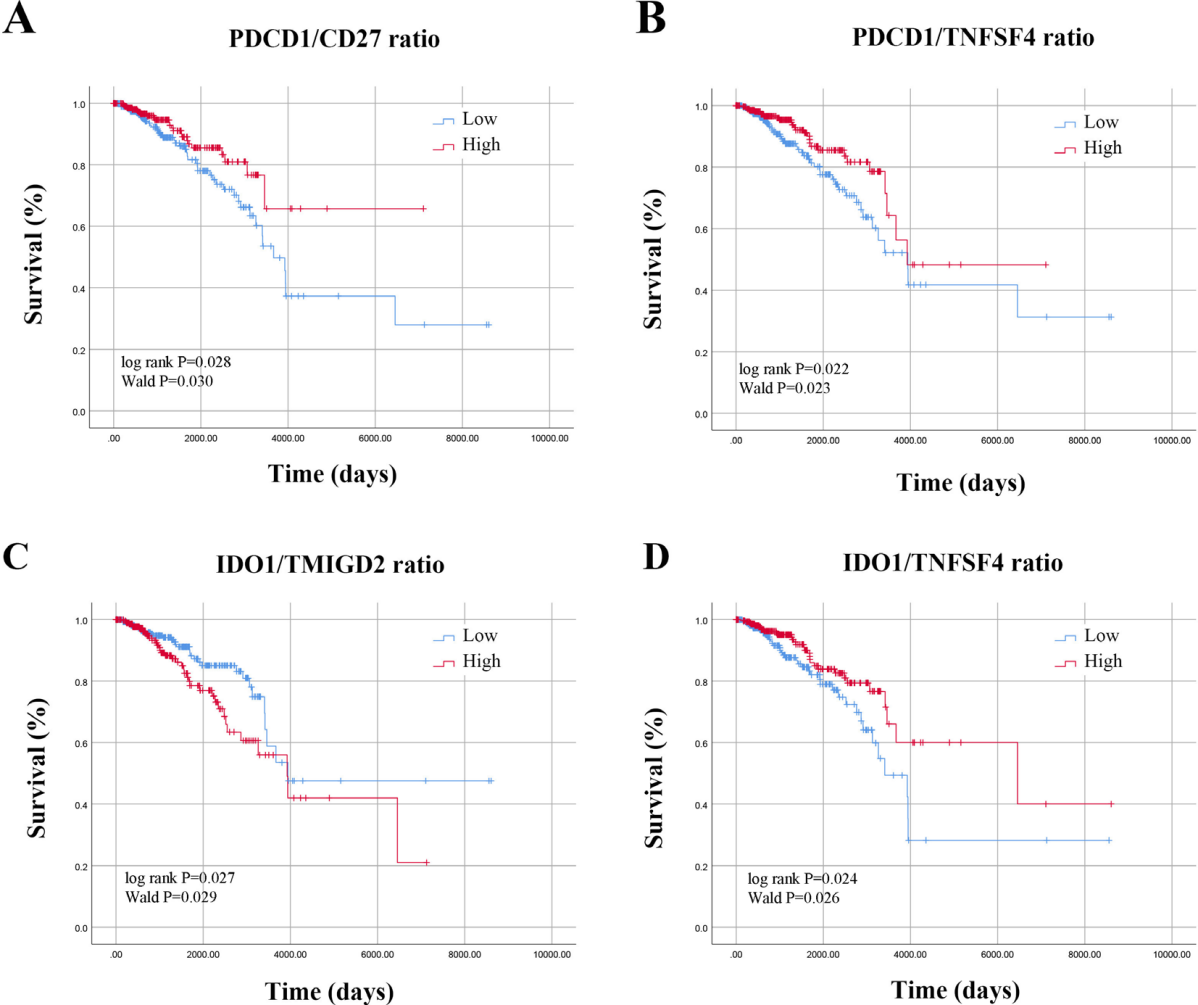
Survival curves based on ISICPRs. **(A)** PDCD1/CD27 ratio; **(B)** PDCD1/TNFSF4 ratio; **(C)** IDO1/TMIGD2 ratio; **(D)** IDO1/TNFSF4 ratio.

Based on the median values of ISICPRs, the cohort was divided into two groups: low-ratio and high-ratio. KM survival analysis showed that the PDCD1/CD27 ratio, PDCD1/TNFSF4 ratio, IDO1/TMIGD2 ratio, and IDO1/TNFSF4 ratio were significantly associated with the survival of BRCA patients (**Figures 3A–D**, P < 0.05). The other 14 candidate ISICPRs showed no association with clinical outcomes (**Supplementary Figure 1**).

**Figure 4:**
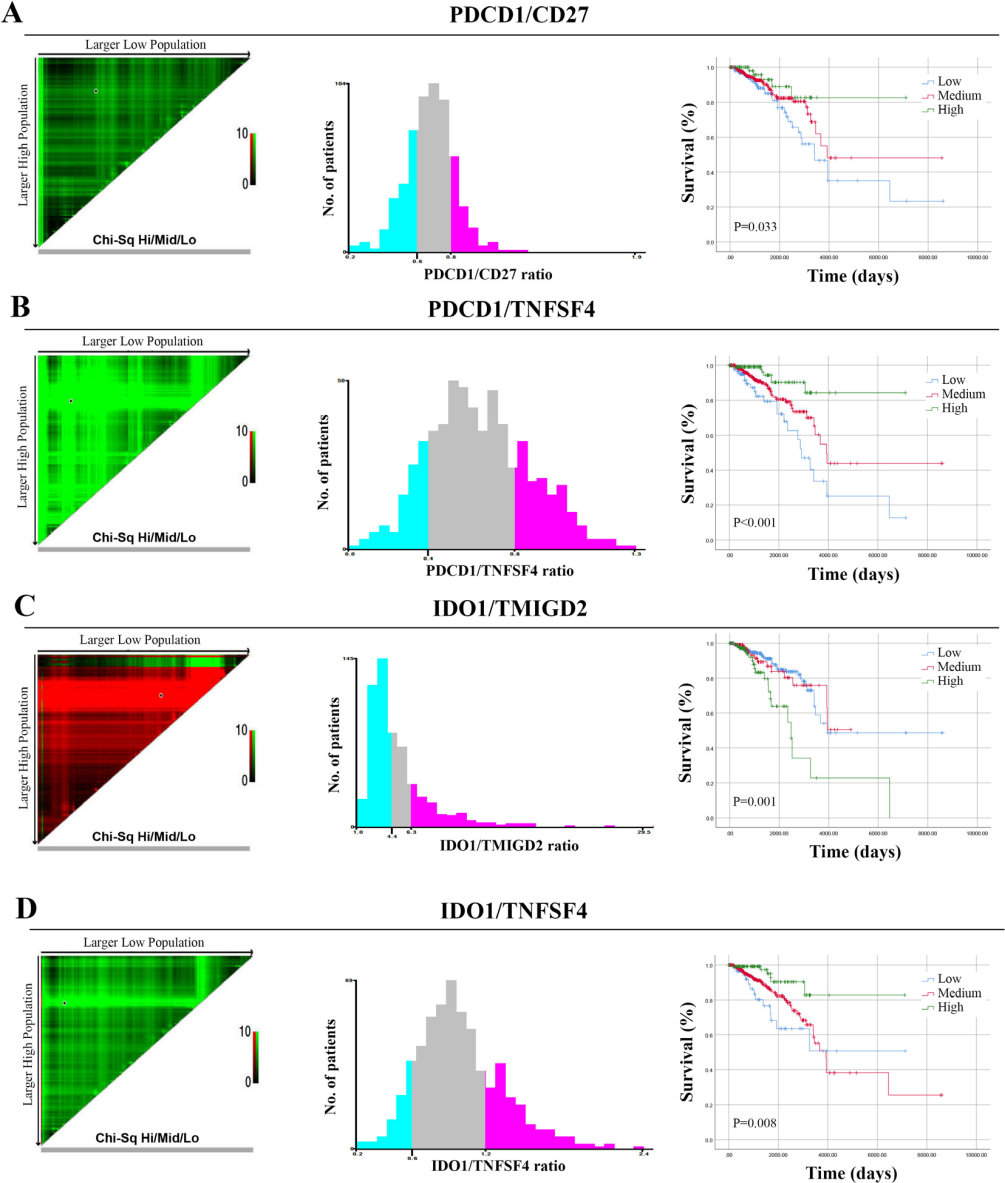
Optimal cut-off values for ISICPRs. **(A)** PDCD1/CD27 ratio; **(B)** PDCD1/TNFSF4 ratio; **(C)** IDO1/TMIGD2 ratio; **(D)** IDO1/TNFSF4 ratio.

Next, in light of the prognostic significance of these ISICPRs, we further analyzed their optimal cutoff values using X-tile software. The cohort was further divided into three groups based on these optimal cut-off values: low-ratio, medium-ratio, and high-ratio groups. For the PDCD1/CD27 ratio, the optimal cut-off values were 0.6 and 0.8 (**Figure 4A**). The low-ratio group had worse outcomes compared to the high-ratio group (**Figure 4A**, P=0.016). For the PDCD1/TNFSF4 ratio, the optimal cut-off values were 0.4 and 0.8 (**Figure 4B**). The low-ratio group exhibited significantly shorter OS compared to the high-ratio group (**Figure 4B**, P < 0.001). For the IDO1/TMIGD2 ratio, the optimal cut-off values were 4.4 and 6.3 (**Figure 4C**). The high-ratio group had a worse prognosis than both the low- and medium-ratio groups (**Figure 4C**, P < 0.001). Finally, for the IDO1/TNFSF4 ratio, the optimal cut-off values were 0.6 and 1.2 (**Figure 4D**). Both the low- and medium-ratio groups had worse prognoses compared to the high-ratio group (**Figure 4D**, P < 0.01).

### IDO1/TMIGD2 ratio

**Figure 5:**
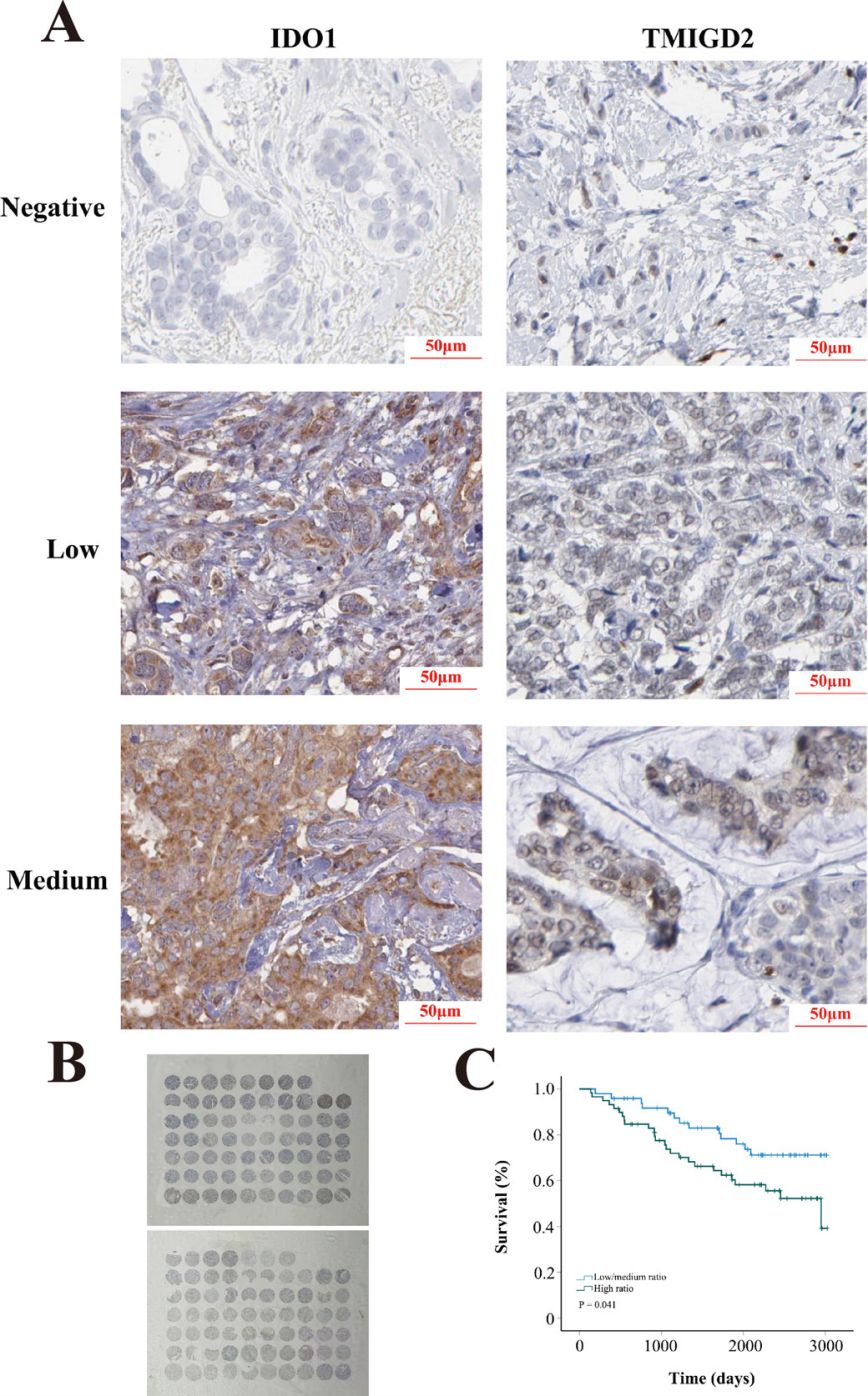
Tissue microarray. **(A)** Representative images of IDO1 and TMIGD2 staining. **(B)** Slide images. **(C)** KM survival analysis.

Univariate analysis demonstrated that age, the PDCD1/CD27 ratio (low vs. high), the PDCD1/TNFSF4 ratio, the IDO1/TMIGD2 ratio (low vs. high), the IDO1/TNFSF4 ratio (low vs. high), neoadjuvant treatment (no vs. yes), and TNM stage significantly influenced prognosis (**Table 2**). Notably, as TNM stage increased, there was a progressive rise in mortality risk for BRCA patients. Subsequently, all factors identified from the univariate analysis were included in the multivariate analysis: age, PDCD1/CD27 ratio, PDCD1/TNFSF4 ratio, IDO1/TMIGD2 ratio, IDO1/TNFSF4 ratio, neoadjuvant treatment, initial weight of tumor, ER status, PR status, HER2 status, and TNM stage. Multivariate analysis revealed that age, the IDO1/TMIGD2 ratio (low/medium vs. high), PR status, neoadjuvant treatment (no vs. yes), and TNM stage were independent prognostic factors (**Table 3**). In particular, a high IDO1/TMIGD2 ratio emerged as a risk factor for BRCA. We further used the tissue microarray to validate the prognostic value of IDO1/TMIGD2 ratio (**Figures 5A, B**), and found that patients with a high IDO1/TMIGD2 ratio also had worse outcomes (**Figure 5C**).

**Table 2:** Univariate analysis for BRCA patients.

Variables	HR	95% CI	P-value
Age, yrs			
< 60	Reference		
≥ 60	2.681	1.664-4.321	<0.001***
PDCD1/CD27 ratio			0.039*
Low	Reference		
Medium	0.640	0.393-1.043	0.073
High	0.338	0.131-0.873	0.025*
PDCD1/TNFSF4 ratio			<0.001***
Low	Reference		
Medium	0.524	0.312-0.881	0.015*
High	0.185	0.079-0.434	<0.001**
IDO1/TMIGD2 ratio			0.001**
Low	Reference		
Medium	1.102	0.585-2.074	0.765
High	2.790	1.606-4.847	<0.001***
IDO1/TNFSF4 ratio			0.013*
Low	Reference		
Medium	0.722	0.392-1.327	0.294
High	0.236	0.089-0.623	0.004**
ER status			
Negative	Reference		
Positive	1.096	0.619-1.938	0.754
HER2 status			
Negative	Reference		
Positive	0.827	0.411-1.666	0.595
PR status			
Negative	Reference		
Positive	0.866	0.534-1.406	0.561
TNM stage			<0.001***
I	Reference		
II	1.257	0.595-2.657	0.549
III	2.320	1.072-5.022	0.033*
IV	9.054	3.649-22.468	<0.001***
History of neoadjuvant treatment			
No	Reference		
Yes	9.036	2.721-30.005	<0.001***
Initial weight of tumor, g			
Median (range)	1.000	0.999-1.001	0.434

ER, estrogen receptor; PR, progesterone receptor; HER2, human epidermal growth factor receptor-2; TNM, tumor-node-metastasis. yrs, years; BRCA, breast cancer; HR, hazard ratio; 95% CI, confidence interval. *P < 0.05; **P < 0.01; ***P < 0.001.

**Table 3:** Multivariate analysis for BRCA patients.

Variables	HR	95% CI	P-value
Age, yrs			
< 60	Reference		
≥ 60	3.692	2.052-6.642	<0.001***
PDCD1/CD27 ratio			0.277
Low	Reference		
Medium	1.635	0.824-3.245	0.160
High	0.958	0.266-3.448	0.948
PDCD1/TNFSF4 ratio			0.092
Low	Reference		
Medium	0.625	0.302-1.297	0.207
High	0.202	0.064-1.006	0.053
IDO1/TMIGD2 ratio			0.065
Low	Reference		
Medium	0.953	0.474-1.916	0.892
High	2.040	1.080-3.854	0.028*
IDO1/TNFSF4 ratio			0.157
Low	Reference		
Medium	0.600	0.290-1.240	0.168
High	0.265	0.066-1.066	0.061
ER status			
Negative	Reference		
Positive	0.583	0.261-1.302	0.188
HER2 status			
Negative	Reference		
Positive	0.563	0.260-1.221	0.146
PR status			
Negative	Reference		
Positive	0.407	0.203-0.815	0.011*
TNM stage			0.022*
I	Reference		
II	1.276	0.569-2.862	0.554
III	2.201	0.971-4.990	0.059
IV	3.816	1.363-10.683	0.011*
History of neoadjuvant treatment			
No	Reference		
Yes	8.913	2.359-33.672	0.001**
Initial weight of tumor			
Median (range)	1.001	1.000-1.002	0.203

ER, estrogen receptor; PR, progesterone receptor; HER2, human epidermal growth factor receptor-2; TNM, tumor-node-metastasis. yrs, years; BRCA, breast cancer; HR, hazard ratio; 95% CI, confidence interval. *P < 0.05; **P < 0.01; ***P < 0.001.

Patients with a high IDO1/TMIGD2 ratio tended to be older and had a higher percentage of HER2-positive tumors (**Table 4**). There were no significant differences observed regarding gender, ER status, PR status, neoadjuvant treatment, initial weight of tumor, or TNM stage.

**Table 4:** Comparison between the low/medium IDO1/TMIGD2 ratio and high IDO1/TMIGD2 ratio.

Variables	Low/medium IDO1/TMIGD2 ratio	High IDO1/TMIGD2 ratio	P-value
Total	462	124	
Gender			0.463
Male	4	2	
Female	458	122	
Age, yrs			0.014*
< 60	273	58	
≥ 60	189	66	
ER status			0.061
Positive	351	104	
Negative	111	20	
PR status			0.577
Positive	312	87	
Negative	150	37	
HER2 status			0.043*
Positive	63	26	
Negative	399	98	
TNM stage			0.053
I	87	17	
II	247	83	
III	118	21	
IV	10	3	
History of neoadjuvant treatment			1.000
No	454	123	
Yes	7	1	
Initial weight of tumor, g	304.194 ± 251.931	282.523 ± 203.199	0.406

ER, estrogen receptor; PR, progesterone receptor; HER2, human epidermal growth factor receptor-2; TNM, tumor-node-metastasis. yrs, years; g, gram.

**Figure 6:**
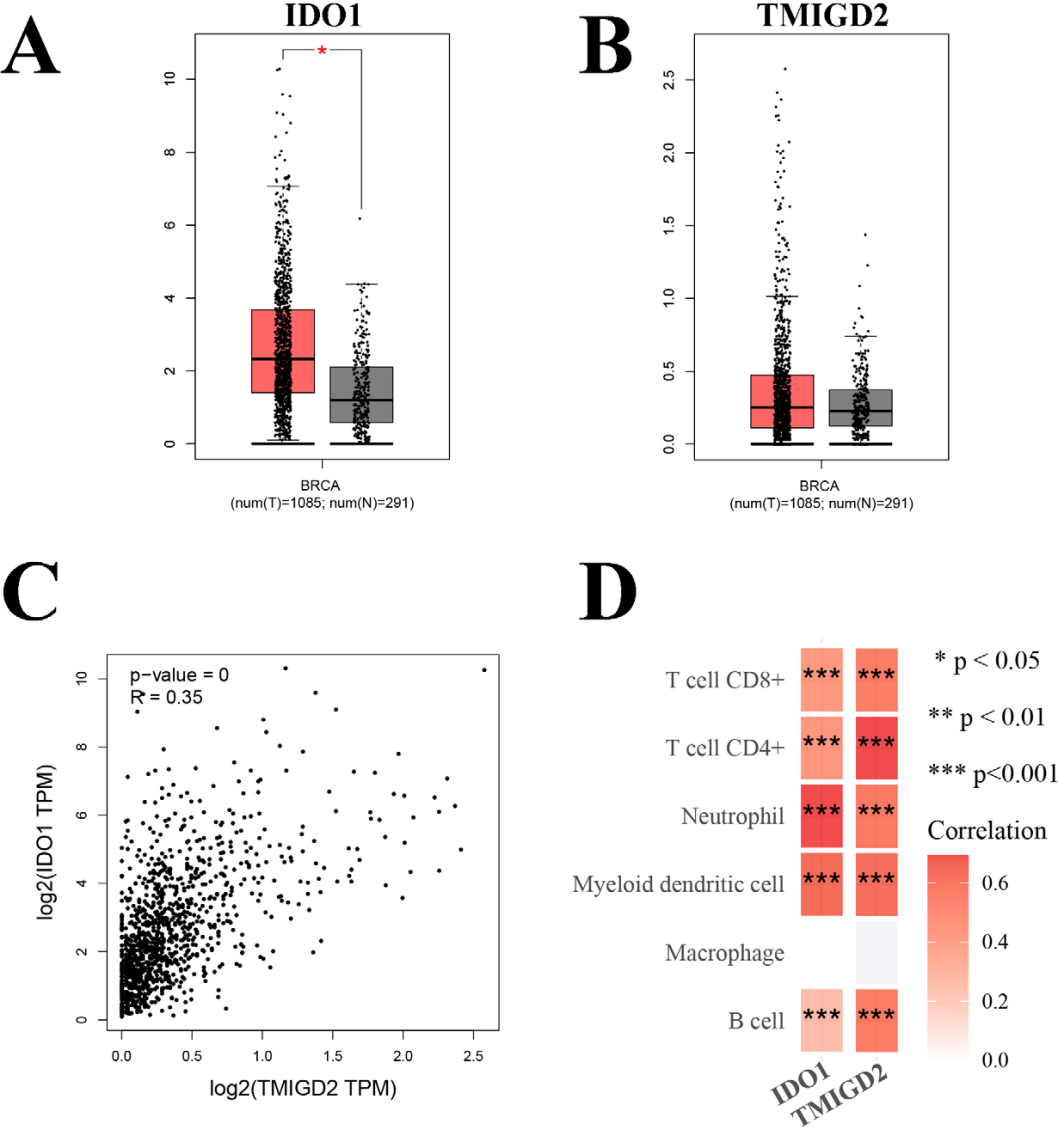
Correlation analysis. **(A)** Comparison of the expression levels of IDO1 in BRCA tissues and normal tissues; **(B)** Comparison of the expression levels of TMIGD2 in BRCA tissues and normal tissues; **(C)** Correlation analysis between IDO1 and TMIGD2; **(D)** Immune correlation analysis of IDO1 and TMIGD2. *P < 0.05; **P < 0.01; ***P < 0.001.

Given the close association between ICPs and immune cells, we further explored the relationship between IDO1 and TMIGD2 with immune cell infiltration. Compared to normal tissues, IDO1 expression was significantly elevated in BRCA tissues, while TMIGD2 expression did not show any significant difference (**Figures 6A, B**). Correlation analysis indicated a significantly positive correlation between IDO1 and TMIGD2 (**Figure 6C**, R = 0.35, P < 0.001). Immune correlation analysis showed that both IDO1 and TMIGD2 were positively correlated with almost all major immune cell subtypes, except macrophages (**Figure 6D**, P < 0.001).

## Discussion

Research on immunotherapies in BRCA began relatively late, as BRCA was long considered a non-immunogenic neoplasm. However, a significant presence of tumor-infiltrating lymphocytes has been observed in BRCA tumors (17). The imbalance of various immunologic factors contributes to the immune evasion of malignant cells. In this study, we investigated the prognostic value of common ISICPRs and identified the IDO1/TMIGD2 ratio as an independent prognostic factor for BRCA. ISICPRs could reflect the balance of the immune system more accurately compared to immune checkpoints. These findings suggest that the disproportion between inhibitory and stimulatory ICPs may be a potential mechanism underlying BRCA initiation and progression, and that ISICPRs could serve as novel prognostic biomarkers in clinical practice. Through accurately predicting clinical outcomes of BRCA patients, clinicians can develop more personalized treatment strategies for individuals, potentially improving survival rates and reducing unnecessary treatments.

IDO1 (full name: indoleamine 2,3-dioxygenase 1) is upregulated in most malignancies, including BRCA, and plays a role in various pathophysiological processes (18, 19). As an immune modulator, IDO1 not only inhibits the function of effector T and NK cells but also activates myeloid-derived suppressor cells and promotes their differentiation (20). In triple-negative BRCA, elevated IDO1 levels were associated with regulatory T cell infiltration and worse survival outcomes (21). Targeting IDO1 selectively has demonstrated enhanced anti-tumor effects when combined with EpCAM/CD3-bispecific antibodies in BRCA with high IDO1 expression, underscoring its clinical importance (22). In this study, we found that the mRNA expression levels of IDO1 were higher than those of TMIGD2 in nearly all patients, indicating that both the activation of inhibitory immune pathways and the weakening of stimulatory pathways promote tumor immune evasion. Notably, the proportion of HER2-positive BRCA was relatively higher in patients with a high IDO1/TMIGD2 ratio. This could be attributed to the enhanced immunosuppressive features of HER2-positive BRCA (23–25). Future clinical trials could use the IDO1/TMIGD2 ratio as a reliable stratification biomarker. For patients with a high ratio, dual targeting of IDO1 and TMIGD2 may be a more effective therapeutic strategy.

TMIGD2, transmembrane and immunoglobulin domain containing 2, is mainly expressed in endothelial and epithelial cells (26). Zhu et al. (27) reported that TMIGD2 was expressed by all naive T cells, though chronic antigen exposure resulted in the loss of TMIGD2 expression in many T cells. The restricted expression pattern of TMIGD2 weakens the stimulatory immune pathways, contributing to tumor immune evasion, which may explain the relatively low TMIGD2 expression levels observed in BRCA.

While other ISICPRs such as PDCD1/CD27 showed significant associations with survival in the univariate analysis, they failed to achieve statistical significance as independent prognostic factors in the multivariate analysis. This may be related to multiple factors. For example, the specific tumor microenvironment of BRCA might limit the significance of their combined effects. Additionally, ISICPRs may be influenced by various confounding factors (e.g. tumor stage and hormone receptor status), and their independent prognostic value may be weakened after adjusting for these factors.

Age has been associated with immunity decline (28, 29). A recent large-scale study indicated that aging leads to widespread up-regulation of ICPs in cancer patients (30). In our study, BRCA patients with a high IDO1/TMIGD2 ratio were generally older. This could be related to the activation of the IDO1-KYN-AhR pathway, which is known to increase with the aging process (31). Given the significant impact of age on immune status in elderly cancer patients, it is essential to consider age as a variable when defining inclusion and exclusion criteria for clinical trials.

There were several limitations to this study. First, all data were retrospective, which may introduce selection bias. The TCGA dataset only includes data from Western populations, which may limit the generalizability of our findings to other ethnic groups. Moreover, a considerable amount of gene data on immune checkpoints is missing due to the retrospective nature of the data. Prospective validation studies with larger sample sizes would strengthen our conclusions. For example, prospective and multicenter clinical studies should be conducted in China to better account for the genetic, physiological, and environmental differences between Chinese and Western populations. Second, the role of ISICPRs in specific pathological subtypes was not evaluated, which limited the applicability of the findings for precision medicine. Third, we did not include other clinical or biological parameters, including immune cell infiltration, tumor markers, disease-specific survival or progression-free survival, which could affect the clinical relevance of ISICPRs. Fourth, although a variety of potential confounding factors were included in our study, these factors may not be comprehensive. Finally, the limited number of male participants may reduce the generalizability of our findings.

In summary, we found the IDO1/TMIGD2 ratio to be an independent prognostic factor for BRCA. On one hand, this novel biomarker could contribute to improved management of BRCA patients. On the other hand, the ISICPR was demonstrated to be a promising indicator with high clinical value, warranting further exploration in other cancer types.

## Data Availability

Publicly available datasets were analyzed in this study. This data can be found here: https://tcga-data.nci.nih.gov/tcga/.

## Supplementary material

Supplementary Figure 1 ISICPR candidates unrelated to BRCA outcomes.
