# Supplementary figures and images for: Prognostic significance of the inhibitory-to-stimulatory immune checkpoint ratio in patients with breast cancer
Source: Front Oncol. 2025 Feb 21;15:1524861. doi: 10.3389/fonc.2025.1524861 (PMC11885121; doi:10.3389/fonc.2025.1524861)

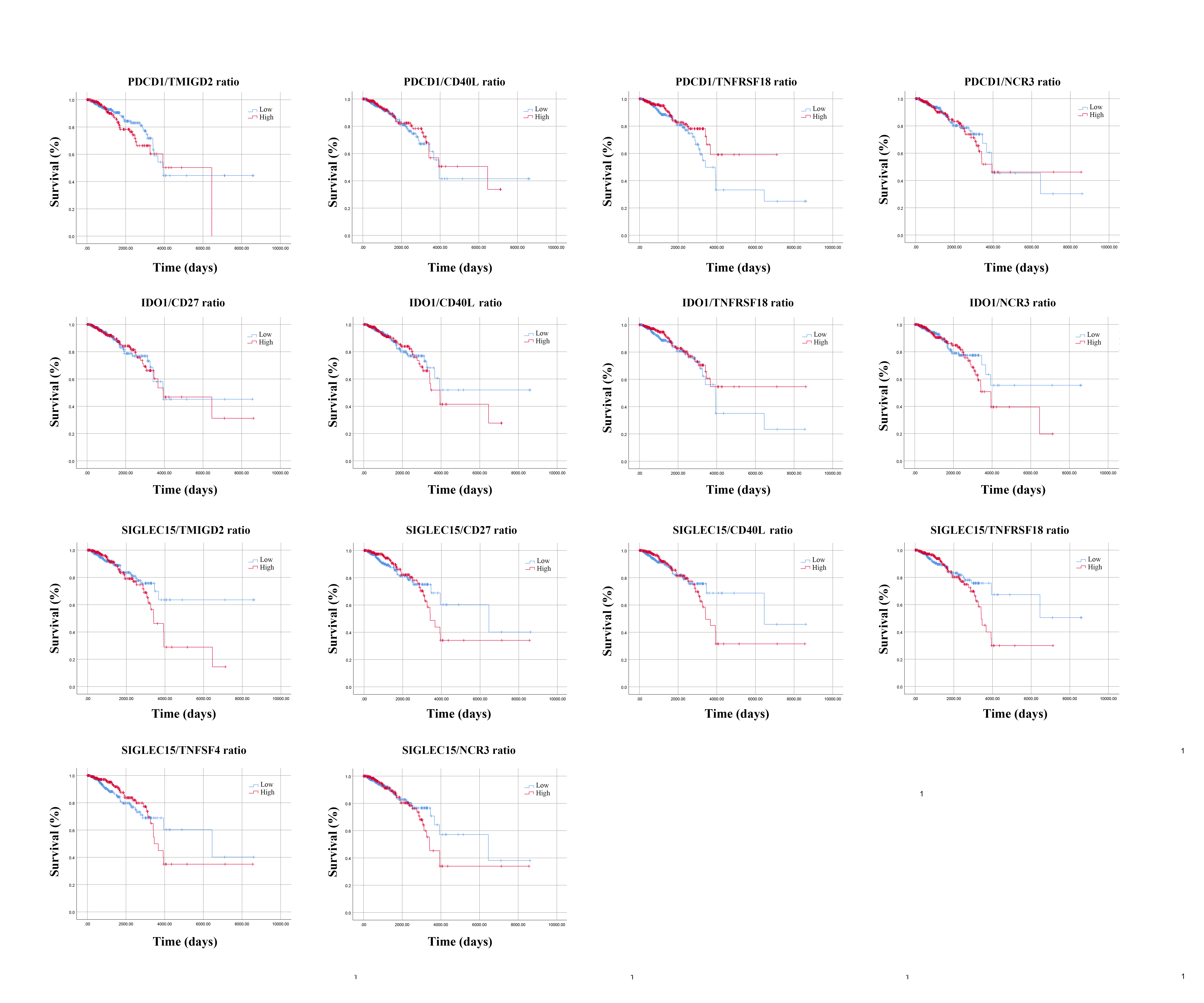

Supplement: Supplementary Figure 1 — ISICPR candidates unrelated to BRCA outcomes. [file Image1.jpeg]
